# MicroRNA-451 as an Early Predictor of Chronic Kidney Disease in Diabetic Nephropathy

**DOI:** 10.1155/2020/8075376

**Published:** 2020-08-14

**Authors:** Mostafa Abdelsalam, A. M. Wahab, Maysaa El Sayed Zaki, Mohamad Motawea

**Affiliations:** ^1^Mansoura Nephrology and Dialysis Unit, Internal Medicine Department, Faculty of Medicine, Mansoura University, Mansoura, Egypt; ^2^Clinical Pathology Department, Faculty of Medicine, Mansoura University, Mansoura, Egypt; ^3^Endocrinology Unit, Internal Medicine Department, Faculty of Medicine, Mansoura University, Mansoura, Egypt

## Abstract

**Background:**

Diabetes mellitus is the leading cause of end-stage renal disease worldwide. Microalbuminuria is the cornerstone for the diagnosis of diabetic nephropathy. However, it is an inadequate marker for early diagnosis. MicroRNAs are not only new and promising markers for early diagnosis but also, but they may also play a role in the prevention of disease progression.

**Methods:**

This study included ninety patients with type 2 DM in addition to 30 control subjects. MicroRNA-451 expression in blood and plasma using real-time PCR was evaluated in addition to the classic diabetic nephropathy markers (serum creatinine, urinary albumin, and eGFR).

**Results:**

There was a significant difference between the studied groups versus control regarding serum creatinine, eGFR, urinary, and plasma microRNA-451 with *p*=0.0001. Patients with eGFR 60 ml/min/1.73 m^2^ showed a significantly higher plasma microRNA-451 (29.6 ± 1.6) and significantly lower urinary microRNA-451 (21 ± 0.9) in comparison to patients with eGFR >60 ml/min/1.73 m^2^ and *p*=0.0001. eGFR showed a positive correlation with urinary microRNA-451 and negative correlation with both plasma microRNA-451 and urinary albumin. Both plasma and urinary microRNA-451 are highly sensitive and specific markers for chronicity in diabetic nephropathy patients with sensitivity of 90.9% and 95.5% and specificity of 67.6% and 95.6%, respectively.

**Conclusion:**

MicroRNA-451 is a promising early biomarker for chronic kidney disease in diabetic nephropathy with high sensitivity and specificity.

## 1. Introduction

There is an increased prevalence of diabetes mellitus (DM) all over the world. The major complications related to DM are attributed to the microvascular and macrovascular affections [1]. Diabetic nephropathy is one of the most common and serious complications in diabetic patients, as well as being the leading cause of end-stage renal disease worldwide [2]. Microalbuminuria is the cornerstone for early diagnosis of diabetic nephropathy. However, many studies have denoted this marker as an inadequate marker for early diagnosis, though it is easy and accessible [3].

Molecular techniques with different accessible detection of biomarkers have been developed to use the new biomarkers as a target for early diagnosis of diabetic nephropathy [4].

MicroRNAs (miRNAs) represent an appeal diagnostic biomarker for many diseases, including renal tumors [5], diabetic nephropathy (DN) [6, 7], and immunoglobulin-A nephropathy [8]. MicroRNAs are a family of short sequences of nucleotides 22–25 long that function by specific binding to the 3′ untranslated regions of their target mRNAs and lead to mRNA degradation or translational repression [9–11].

MicroRNAs play an essential role in regulating cell proliferation, differentiation, apoptosis, neoplastic transformation, and apoptosis. The role of miRNAs in DN development is through the development of renal fibrosis [12]. The studies hypothesized that some miRNAs lead to renal fibrosis through their effects on the transforming growth factor -*β*1 (TGF-*β*1) signal pathway and their impact on epithelial-mesenchymal transformation [13, 14]. The principal function of TGF-*β*1 is the regulation of proliferation in the extracellular matrix, fibroblast, and myofibroblasts [15]. In renal damage, the numbers of TGF-*β*1 receptors increase with the initiation of fibrosis [16]. About twenty-two types of miRNAs were studied in diabetic nephropathy; fifteen were upregulated (miRNA-181b, miRNA-21, miRNA-30, miRNA-194, miRNA-215, and others). At the same time, seven of them were downregulated in the diabetic nephropathy group compared to control groups (miRNA-26a, miRNA-424, miRNA-126, miRNA-574-3p, miR-155, miR-223, and miR-192) [17]. Urinary exosomal miR-451-5p was only studied in diabetic rats as an early and sensitive noninvasive indicator of renal disease [18].

The advantages of the use of miRNAs as a diagnostic biomarker are their stability in blood and urine samples and being noninvasive markers. Therefore, the quantitative measurement of miRNA in plasma and urine samples can be used as a diagnostic marker for monitoring the development of diabetic complications leading to the modification of the treatment with increased quality of life and the survival rates of the patients [19].

To the best of our knowledge, this is the first study that evaluated urinary and blood levels of miRNA-451 as an early biomarker for the diagnosis of diabetic nephropathy and chronic kidney disease in diabetic patients.

## 2. Materials and Methods

A case-control study including 90 diabetic patients recruited from outpatients' clinics from Mansoura University Hospital from January 2017 till November 2018 was conducted, in addition to 30 healthy subjects with normal kidney functions, normal blood glucose levels, and normal renal ultrasonography.

Patients were classified according to the presence of albuminuria into four groups:

- Group 1, including 30 patients with normoalbuminuria and urinary albumin level <30 mcg/mg creatinine
- Group 2, including 30 patients with microalbuminuria and urinary albumin level between 30 and 299 mcg/mg creatinine
- Group 3, including 30 patients with macroalbuminuria and urinary albumin level >300 mcg/mg creatinine
- Group 4, including 30 healthy individuals of the same age and sex

The criteria for inclusion of the participant were age above 18 years with known DM type 2 and who are receiving their regular and routine antidiabetic medications, either orally or as an insulin therapy from the outpatients' clinics of Mansoura university hospitals with normal blood pressure, systolic blood pressure ≤140 mmHg and diastolic blood pressure ≤90 mmHg. Patients with congenital kidney diseases, malignancy, chronic glomerulonephritis, endocrinal diseases other than DM, and eGFR <30 ml/min/1.74 m^2^ and those with any abnormal renal ultrasonography finding were excluded. The Medical Ethical Committee of Mansoura Faculty approved the study. A voluntary informed consent was taken from all patients. Confidentiality and privacy were considered as regard personal, clinical, and laboratory data.

Each subject had a complete medical history recording and comprehensive physical examination.

Patients were reclassified according to the GFR level to the following:

- Group 1, including 68 patients with eGFR >60 ml/min/1.73 m^2^
- Group 2, including 22 patients with eGFR <60 ml/min/1.73 m^2^

## 3. Laboratory Methods

### 3.1. Urine Samples

A urine sample was collected in a clean container from each subject and transported to the laboratory for complete urine analysis, including amount registration, glucose, ketone, creatinine, and albumin measurement. Quantitative measurement of urinary albumin and creatinine was carried out by using biosystem kits (Biosystems S.A., Barcelona); after that, an aliquot of urine was kept at −80°C for measurement of miRNA-451.

### 3.2. Urinary Albumin

The albumin in urine is measured by turbidimetry method as albumin binds to antihuman albumin coating agglutination latex particles. The quantity of albumin was measured at 505 wave length with RA analyzer 50.

### 3.3. Blood Samples

A ten-milliliter blood sample was obtained from each subject after eight hours of fasting and divided into three aliquots. One aliquot was used for complete blood counts by Sysmex system and HBA1c by Stanbio kit (Boerne, Texas, USA, Cat. No. 0350) by the use of quantitative colorimetric method using column chromatography for determination of glycohemoglobin in whole blood. From the other aliquot, serum was separated and subjected to laboratory measurement of serum creatinine, albumin, cholesterol, total triglycerides, high-density lipoprotein (HDL), low-density lipoprotein (LDL), blood urea nitrogen (BUN), and fasting blood glucose level by autoanalyzer Dialab (A-2351 Wr. Neudorf, Austria). Another blood sample was obtained after two hours for postprandial blood glucose levels. The serum was separated from the third aliquot and kept frozen at −80°C for the molecular study of miRNA-451 by a real-time polymerase chain reaction.

### 3.4. eGFR Measurement

MDRD equation was used to calculate the eGFR for each subject:

- eGFR (mL/min/1.73 m^2^) = 175 × (*S*_cr_)^−1.154^ × (age)^−0.203^ × (0.742 if female)

### 3.5. RNA Extraction and Quantification

MicroRNA-451 extraction from the plasma samples was performed by Qiagen miRNA Easy kit and from the urine samples by Norgen Urine microRNA purification kit, following the manufacturers' instructions. MicroRNA-451 concentrations and purity were determined with Nano-Drop 2000 UV spectrophotometer (Thermo Scientific, USA) using the A260/280 nm ratio.

### 3.6. Reverse Transcriptase-Polymerase Chain Reaction (qRT-PCR)

The expression of mature miR-451 was determined by qRT-PCR, as described in detail previously using the TaqMan Human MicroRNA Assay kit (Life Technologies-catalogue number #001141) and the real-time PCR system (Applied Biosystems, Carlsbad, CA, USA) [20]. The endogenous internal control was RNU6B (Life Technologies-catalog number #4440887). All assays were performed in triplicate. Real-time PCR results were recorded as threshold cycle numbers (Ct) and were normalized against an internal control (U6 RNA) and then expressed as fold changes. The miRNA expression was quantified as *δCt* values, where *Ct* = threshold cycle, *δCt* = (*Ct* target microRNA − *Ct* RNU6B).

## 4. Statistical Analysis

Statistical analysis was carried out by the use of SPSS 22. Numerical Data are presented as mean ± SD for normally distributed values. ANOVA analyzed differences between the groups. Pearson's correlation coefficient was employed to test the correlations between different variables. Receiver operating characteristics (ROC) analysis was used to calculate the area under the curve (AUC) for microRNA-451 levels of serum and urine to find the best cutoff values for identifying renal impairment in diabetic patients. All results were considered significant if *p* < 0.05.

## 5. Results

In the current study, patients were classified according to the level of albuminuria into three groups. There was no significant difference between the studied groups as regard age, sex, WBCs, platelets, and HDL (Table 1).

On the other hand, there was a significant difference in the levels of fasting blood glucose, creatinine, BUN, HBA1c, and hemoglobin with *p*=0.0001. Total cholesterol, LDL, and TGs showed a significant difference between the studied group with *p*-value, 0.0001, 0.0001, and 0.002, respectively. The eGFR had a significant difference among the studied groups (*p*=0.0001). Both urinary and plasma microRNA-451 showed a statistically significant difference in different patients' groups compared to the control group (*p*=0.0001). Diabetic patients with macroalbuminuria showed the highest level of plasma microRNA-451 and the lowest level of both urinary microRNA-451 and eGFR (Table 1).

Sixty-eight patients (75.6%) showed eGFR >60 ml/min/1.73 m^2^ and were included in group [1] while twenty-two patients (24.4%) with eGFR between 30 ml/min/1.73 m^2^ and 60 ml/min/1.73 (5 with normoalbuminuria, 7 with microalbuminuria and 10 with macroalbuminuria) were included in group [2] as shown in Table 2.

Comparison between the two groups showed no significant difference regarding age, sex, BUN, fasting blood sugar, HBA1c, total cholesterol, LDL, HDL, TGs, hemoglobin, WBCs, and platelets. On the other hand, there was a significant difference as regard creatinine, albuminuria, plasma microRNA-451, and urinary microRNA-451 with *p*-values 0.0001, 0.035, 0.036, and 0.0001, respectively, as shown in Table 2. In the unshown data due to small sample size, subgroup analysis of patients with eGFR <60 ml/min/1.73 m^2^ (five patients) showed a significant increase in plasma microRNA-451 and decrease in urinary microRNA-451 in comparison to the control group with *p*-values 0.01 and 0.008, respectively.

The eGFR showed a significantly negative correlation with albuminuria and plasma microRNA-451, *r* = −0.491, *p*=0.0001 and *r* = −0.458, *p*=0.0001, respectively, and a positive correlation with urinary microRNA-451 (*r* = 0.262, *p*=0.013) as shown in Table 3.

For the determination of sensitivity and specificity of microRNA-451 in blood and urine as biomarkers for early detection of diabetic nephropathy and chronic kidney disease in our patients, ROC analysis was used. The area under the curve was 0.427 for urinary microRNA-451 and 0.625 for plasma microRNA-451. The sensitivity of plasma microRNA-451 was 90.9% for cutoff 27.5 with a specificity of 67.7%, while the sensitivity of plasma microRNA-451 was 95.5% for cutoff 19.5 with specificity was 95.6%, as shown Table 4.

## 6. Discussion

Diabetes mellitus is the leading cause of end-stage renal disease worldwide [21]. Early detection of diabetic nephropathy is still a challenge. Regular followup urinary albumin is still the cornerstone for early evaluation of renal involvement in diabetic patients. However, there are patients with a decline in renal functions not accompanied by microalbuminuria or increased creatinine levels [22, 23]. In the present study, 16.7% of patients with normoalbuminuria had reduced eGFR, which was in agreement with Krolewski et al., who stated that approximately 10–30% of diabetic patients develop renal impairment before the onset of microalbuminuria or macroalbuminuria [24].

The change in creatinine level, together with increased albumin in urine, appears as an attractive biomarker for the diagnosis of glomerular dysfunction associated with DM. However, these markers have many limitations, such as the tubular affection in DM, which may precede glomerular dysfunction leading to normal serum creatinine and normal level of urine albumin [25].

Therefore, there is a need for new, easily accessible, and noninvasive biomarkers for early detection of diabetic nephropathy. Among these biomarkers is the microRNA expression, which are affected by the pathological conditions involving kidneys [26].

In the present study, plasma microRNA-451 showed a significant increase in patients with DM and mild to moderate end-stage renal disease either with or without albuminuria. These findings were similar to the previous experimental study by Zhang et al. [27, 28].

The increased expression of miR-451 has a protective role in DN that leads to the inhibition of glomerular and mesangial cell proliferation, in both in vivo and in vitro studies. This pathway is mediated through the downregulation of Ywhaz and p38 MAPK signal pathway. These are the target genes for miR-451, which downregulated the expression of Ywhaz (tyrosine3-monooxygenase, tryptophan 5 monooxygenase activation protein, and zeta) gene in the 3′ UTR. The overexpression of miR-451 caused the reduced activities of the two kinases (p38 MAPK and MKK3) and the suppression of Ywhaz [28]. Therefore, this miRNA may be a potential target for intervention and prevention of DN in an early stage.

On the other hand, urinary microRNA-451 in the present study showed a significant reduction in patients with eGFR <60 ml/min/1.73 m^2^ compared to the other group of patients with eGFR >60 ml/min/1.73 m^2^. Our result matched the previous study which reported a reduction of all miRNA levels as they decreased in parallel with the renal functions among patients with end-stage renal disease [29]. The higher levels of microRNA-451 expression in plasma in association with its low expression in urine samples in patients with low eGFR were in agreement with previous studies in diabetic nephropathy patients [26] and hypertension associated with kidney affection [30]. These findings indicate that the expression of microRNA-451 in blood and urine can be an accurate marker for early diagnosis of DN even in the absence of albuminuria.

There was a significant positive correlation between the urinary microRNA-451 level and the eGFR value in association with a negative correlation between the plasma microRNA-451 level and eGFR, which supports the role of microRNA-451 as a predictor of disease progression and the development of renal fibrosis [30]. Previous studies for microRNA-451 revealed an early increase in its level in the tissue of the kidney and later on a decrease in urine, reflecting the increased activity of signal pathways such as MAPK9 and SMAD3 in association with the process of nephropathy development [27, 30]. Therefore, the expression of microRNA-451 in urine might reflect the glomerular filtration rate and renal production of this microRNA.

The question raised either from this study and/or the previous studies was about the use of microRNA-451 as a predictor for the disease progression and a therapeutic approach to prevent the development of DN. This approach can be mediated through miRNA mimics, miRNA expression vectors, miRNA-containing exosomes, and miRNA-inducing natural agents with the restoration of renal-protective miRNAs levels and subsequent protection from DN [31].

The present study has shown that both plasma and urinary microRNA-451 can be used as a sensitive and specific marker for the early diagnosis of DN. The sensitivity of plasma microRNA-451 was 90.9% with a specificity of 67.6%, while the sensitivity of urinary microRNA-451 was 95.5% with specificity 99.6%, which were in agreement with the results of Sayilar et al. [27]. On the other hand, binary logistic regression showed that plasma microRNA-451 was a significant independent predictor for eGFR <60 mL/min/1.73 m^2^ compared to urinary microRNA-451, as shown in Table 5.

## 7. Conclusion

The present study's findings highlight the different expressions of microRNA-451 in blood and urine in different stages of diabetic nephropathy. There is a marked and gradual increase in blood level in different stages, denoting the potential role of this microRNA in the pathogenesis of this condition and its usefulness as an early biomarker for diagnosis. Moreover, the level of urinary microRNA-451 has a gradual reduction, reflecting the role played in the pathogenesis, and makes it a valuable biomarker for early diagnosis of diabetic nephropathy even in the absence of albuminuria. This study has its limitations, including a small sample size and the lack of renal biopsy and pathology, which we think will add a lot of value to our results.

## Figures and Tables

**Table 1:** Comparison of demographic and laboratory data of the studied groups.

Parameters	Normoalbuminuria (30 patients)	Microalbuminuria (30 patients)	Macroalbuminuria (30 patients)	Control (30 individuals)	*p*
Age mean ± SD	53.1 ± 6.2	53.6 ± 5.1	53.6 ± 6.2	50.4 ± 6.9	0.128
Gender					
Male *n* (%)	13 (43.3%)	14 (46.7%)	21 (70%)	17 (56.7%)	
Female *n* (%)	17 (56.7%)	16 (53.3%)	9 (30%)	13 (43.3%)	0.159
Creatinine (mg/dl)	0.9 ± 0.14^abc^	1 ± 0.2^ade^	1.2 ± 0.24^bdf^	0.9 ± 0.2^cef^	0.0001
BUN (mg/dl)	29 ± 1.4^abc^	27.7 ± 2.2^ade^	59 ± 4.3^bdf^	27 ± 2^cef^	0.0001
FBS (mg/dl)	150 ± 34^a^	147 ± 20^b^	136 ± 34^c^	89 ± 9^abc^	0.0001
HBA1c (%)	8.9 ± 1.8^a^	9.2 ± 1.8^b^	9.3 ± 2^c^	4.3 ± 0.6^abc^	0.0001
Hemoglobin (gm/dl)	11.1 ± 1^a^	11 ± 1^b^	10.6 ± 2.1^abc^	12.9 ± 1.4^c^	0.0001
WBCs (mm^3^)	6.4 ± 1.6	6.6 ± 1.6	6.3 ± 1.9	8.9 ± 2	0.097
Platelets (mm^3^)	240 ± 25	232 ± 33	223 ± 37	225 ± 42	0.224
Cholesterol (mg/dl)	202 ± 9^a^	204 ± 14^b^	196 ± 10^c^	172 ± 15^abc^	0.0001
LDL (mg/dl)	119 ± 6^a^	121 ± 7^b^	118 ± 6^c^	109 ± 20^abc^	0.0001
HDL (mg/dl)	45 ± 4^a^	44 ± 3^b^	44 ± 5^c^	44±4^abc^	0.86
TGs (mg/dl)	127 ± 9^a^	125 ± 6^b^	134 ± 13^abc^	126 ± 11^c^	0.002
eGFR <60 ml/min/1.73 m^2^*n* (%)	5 (16.7%)	7 (23.3%)	10 (33.3%)	—	0.32
eGFR ml/min/1.73 m^2^	70.2 ± 14.6^ade^	63.4 ± 9^bdf^	61.2 ± 10.7^cef^	112 ± 11.2^abc^	0.0001
U-microRNA-451	24.3 ± 2.8^ade^	22.9 ± 1.6^bdf^	21.1 ± 1.5^cef^	32 ± 2^abc^	0.0001
P-microRNA-451	27.4 ± 0.8^ade^	28.4 ± 1^bdf^	31 ± 1^cef^	25.8 ± 1.8^abc^	0.0001

(i) Probability of chi square *t*-test was used for qualitative data and values of its variables are expressed as *n* (%) number (percentage.). (ii) Probability of one-way “ANOVA” between the four groups with subsequent post hoc analysis if significant was used for numerical data and values of its variables are expressed as mean ± standard deviation. (iii) abc: similar letters indicate significant value and ^*∗*^ indicates *p* < 0.05. (iv) FBS: fasting blood sugar; U-microRNA-451: urinary microRNA-451; P-microRNA-451: plasma microRNA-451.

**Table 2:** Comparison of demographic and laboratory data between patients' subgroups.

Parameters	Group (1) eGFR >60 ml/min/1.73 m^2^	Group (2) eGFR <60 ml/min/1.73 m^2^	*p*
Number (%)	68 (75.6%)	22 (24.4%)	—
Age	53 ± 5.8	54.8 ± 6	0.22
Sex			
Male, *n* (%)	39 (57.4%)	9 (41%)	
Female, *n* (%)	29 (42.6%)	13 (59%)	0.22
Creatinine (mg/dl)	1 ± 0.2	1.3 ± 0.2	0.0001
eGFR ml/min/1.73 m^2^	78.4 ± 15.8	51.8 ± 6.4	0.0001
BUN (mg/dl)	31 ± 11	44 ± 14	0.22
Fasting blood glucose (mg/dl)	146 ± 33	141 ± 20	0.36
HBA1c (%)	9 ± 1.8	9.6 ± 2	0.18
Cholesterol (mg/dl)	200 ± 13	203 ± 8	0.35
LDL (mg/dl)	119.7 ± 5.9	117.5 ± 72	0.2
Triglycerides (mg/dl)	128.8 ± 10	127.8 ± 12.1	0.7
HDL (mg/dl)	44.2 ± 3.8	44.1 ± 4.5	0.9
Hemoglobin (gm/dl)	11.8 ± 1.7	10.8 ± 1.8	0.25
WBCs (mm^3^)	6.8 ± 1.7	7 ± 1.5	0.8
Platelets (mm^3^)	233.4 ± 33.6	226.7 ± 28.7	0.37
Albuminuria mcg/mg creatinine	185 (15–670)	250 (19–900)	0.035
Urine microRNA-451	23.9 ± 2.8	21 ± 0.9	0.0001
Plasma microRNA-451	28.7 ± 1.7	29.6 ± 1.6	0.036

(i) Probability of Mann–Whitney U-test for nonparametric data and values of its variables are expressed as median (range). (ii) Probability of chi square *t*-test for qualitative data and values of its variables are expressed as *n* (%) number (percentage). (iii) Probability of independent sample *T*–test for parametric data and values of its variables are expressed as mean ± standard deviation.

**Table 3:** Correlation between eGFR, albumin in urine, and microRNA-451 in urine and in blood in all diabetic patients.

Parameter	eGFR
*Plasma microRNA-451*	
Pearson correlation	−0.458-^*∗∗*^
Sig. (two-tailed)	0.000

*Urinary microRNA-451*	
Pearson correlation	0.262^*∗*^
Sig. (two-tailed)	0.013

*Albuminuria*	
Pearson correlation	−0.491-^*∗∗*^
Sig. (two-tailed)	0.000

^*∗∗*^Correlation is significant at the 0.01 level (two-tailed). ^*∗*^Correlation is significant at the 0.05 level (two-tailed).

**Table 4:** Area under curve for microRNA-451 in blood and urine.

Test result variable(s)	Cutoff	Sensitivity (%)	Specificity (%)	Area	Asymptotic 95% confidence interval	
					Lower bound	Upper bound
Plasma microRNA-451	27.5	90.9	67.6	0.625	0.529	0.774
Urinary microRNA-451	19.5	95.5	95.6	0.427	0.295	0.56

**Table 5:** Binary logistic regression.

Variables in the equation								
	B	S.E.	Wald	d*f*	Sig.	Exp (B)	95% C.I. for exp. (B)	
							Lower	Upper
Step 1a								
Urinemicro	0.019	0.115	0.028	1	0.868	1.019	0.813	1.278
Microplasma	0.318	0.155	4.184	1	0.041	1.374	1.013	1.863
Constant	−10.779-	6.120	3.103	1	0.078	0.000		

## Data Availability

All the data used to support the finding of this study are included in the manuscript and in the supplementary materials.

## Supplementary Materials

Supplementary Materials The authors have attached a single supplementary file that includes some of their patients' data. The authors are responsible for providing the final supplementary materials files that will be published along with the article.
